# (2*E*)-2-Benzyl­idene-4-ethyl-3,4-dihydro­naphthalen-1(2*H*)-one

**DOI:** 10.1107/S1600536811022793

**Published:** 2011-06-18

**Authors:** Mohamed Akhazzane, Hafid Zouihri, A. Kella Bennani, Abdelali Kerbal, Ghali Al Houari

**Affiliations:** a Laboratoire de Chimie Organique, Faculté des Sciences Dhar el Mahraz, Université Sidi Mohammed Ben Abdellah, Fès, Morocco; b Laboratoires de Diffraction des Rayons X, Centre National pour la Recherche, Scientifique et Technique, Rabat, Morocco

## Abstract

In the title compound, C_19_H_18_O, the exocyclic C=C double bond has an *E* configuration. The ethyl substituent on the cyclo­hexa­none ring is in an axial position. The cyclo­hexa­none ring adopts a half-chair conformation, presumably due to conjugation in the benzene ring.

## Related literature

For general background to dipolar-1,3 cyclo­addition reactions, see: Bennani *et al.* (2007 ▶); Kerbal *et al.* (1988 ▶); Al Houari *et al.* (2008 ▶). For a related structure, see: Akhazzane *et al.* (2010 ▶). For puckering parameters, see: Cremer & Pople (1975 ▶).
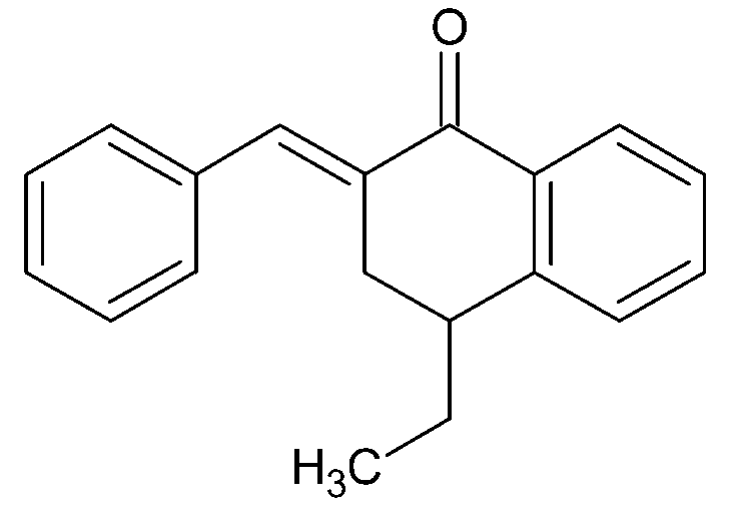


## Experimental

### 

#### Crystal data

C_19_H_18_O
*M* *_r_* = 262.33
Monoclinic, 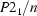

*a* = 11.7997 (8) Å
*b* = 8.9020 (6) Å
*c* = 13.9912 (9) Å
β = 94.214 (4)°
*V* = 1465.68 (17) Å^3^
*Z* = 4
Mo *K*α radiation
μ = 0.07 mm^−1^
*T* = 296 K
0.24 × 0.13 × 0.10 mm

#### Data collection

Bruker APEXII CCD detector diffractometer
12738 measured reflections
2870 independent reflections
1776 reflections with *I* > 2σ(*I*)
*R*_int_ = 0.039

#### Refinement

*R*[*F* ^2^ > 2σ(*F* ^2^)] = 0.047
*wR*(*F* ^2^) = 0.139
*S* = 1.06
2870 reflections
182 parameters
H-atom parameters constrained
Δρ_max_ = 0.15 e Å^−3^
Δρ_min_ = −0.15 e Å^−3^

### 

Data collection: *APEX2* (Bruker, 2005 ▶); cell refinement: *SAINT* (Bruker, 2005 ▶); data reduction: *SAINT*; program(s) used to solve structure: *SHELXS97* (Sheldrick, 2008 ▶); program(s) used to refine structure: *SHELXL97* (Sheldrick, 2008 ▶); molecular graphics: *PLATON* (Spek, 2009 ▶); software used to prepare material for publication: *publCIF* (Westrip, 2010 ▶).

## Supplementary Material

Crystal structure: contains datablock(s) I, global. DOI: 10.1107/S1600536811022793/fj2430sup1.cif

Structure factors: contains datablock(s) I. DOI: 10.1107/S1600536811022793/fj2430Isup2.hkl

Supplementary material file. DOI: 10.1107/S1600536811022793/fj2430Isup3.cml

Additional supplementary materials:  crystallographic information; 3D view; checkCIF report

## Figures and Tables

**Fig. 1.**
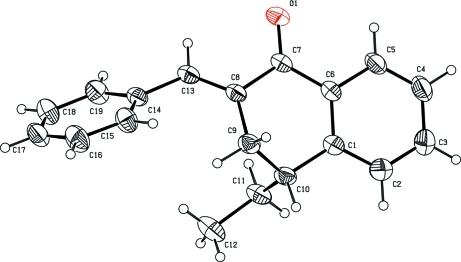
Molecular view of the title compound showing the atom-labeling scheme. Displacement ellipsoids are drawn at the 30% probability level. H atoms are represented as small spheres of arbitrary radii.

## supplementary crystallographic information

### Comment

Knowledge of the configuration and conformation of the title compound is necessary to understand its behaviour in dipolar-1,3 cycloaddition reactions [Bennani, *et al.* (2007) and Al Houari *et al.* (2008)]. To confirm the E configuration of the exocyclic C=C double bond, an X-ray crystal structure determination has been carried out.

The cyclohexanone ring in the dihydronaphthalene fused-ring system adopts a half-chair conformation, presumably due to conjugation of the planar annulated benzo ring,, with the puckering parameters of: Q(2) = 0.403 (2) Å, Phi(2) = 75.0 (3)°, Q(3) = -0.255 (2) Å (Cremer & Pople, 1975). The dihedral angle between the benzene ring and the napthyl ring system is 64.87 (9)°.

In the title compound, as shown in Fig. 1, all bond lengths and angles are normal and comparable with those reported for the related structure [Akhazzane *et al.*, (2010)].

### Experimental

The synthesis of (2E)-2-benzylidene-4-ethyl-3,4-dihydronaphthalen-1(2*H*)-onewas achieved using the method reported by Kerbal and al. [Kerbal *et al.* (1988)]. *i.e.* by a condensation of *para* tolylaldehyde with 4-ethyl-3,4-dihydronaphthalen-1(2*H*)-one in an alkaline medium in methanol.

### Refinement

The H atoms bound to C were treated as riding with their parent atoms [C—H distances are 0.93Å for CH groups with *U*_iso_(H) = 1.2 *U*_eq_(C), and 0.97 Å for CH3 groups with *U*_iso_(H) = 1.5 *U*_eq_(C)].

### Crystal data

**Table:**

C_19_H_18_O	*F*(000) = 560
*M**_r_* = 262.33	*D*_x_ = 1.189 Mg m^−^^3^
Monoclinic, *P*2_1_/*n*	Mo *K*α radiation, λ = 0.71073 Å
Hall symbol: -P 2yn	Cell parameters from 317 reflections
*a* = 11.7997 (8) Å	θ = 2.3–25.7°
*b* = 8.9020 (6) Å	µ = 0.07 mm^−^^1^
*c* = 13.9912 (9) Å	*T* = 296 K
β = 94.214 (4)°	Prism, colourless
*V* = 1465.68 (17) Å^3^	0.24 × 0.13 × 0.10 mm
*Z* = 4	

### Data collection

**Table:**

Bruker APEXII CCD detector diffractometer	1776 reflections with *I* > 2σ(*I*)
Radiation source: fine-focus sealed tube	*R*_int_ = 0.039
graphite	θ_max_ = 26.0°, θ_min_ = 2.2°
ω and φ scans	*h* = −14→14
12738 measured reflections	*k* = −10→10
2870 independent reflections	*l* = −17→17

### Refinement

**Table:**

Refinement on *F*^2^	Primary atom site location: structure-invariant direct methods
Least-squares matrix: full	Secondary atom site location: difference Fourier map
*R*[*F*^2^ > 2σ(*F*^2^)] = 0.047	Hydrogen site location: inferred from neighbouring sites
*wR*(*F*^2^) = 0.139	H-atom parameters constrained
*S* = 1.06	*w* = 1/[σ^2^(*F*_o_^2^) + (0.0502*P*)^2^ + 0.4678*P*] where *P* = (*F*_o_^2^ + 2*F*_c_^2^)/3
2870 reflections	(Δ/σ)_max_ < 0.001
182 parameters	Δρ_max_ = 0.15 e Å^−^^3^
0 restraints	Δρ_min_ = −0.15 e Å^−^^3^

### Special details

**Table:**

Geometry. All e.s.d.'s (except the e.s.d. in the dihedral angle between two l.s. planes) are estimated using the full covariance matrix. The cell e.s.d.'s are taken into account individually in the estimation of e.s.d.'s in distances, angles and torsion angles; correlations between e.s.d.'s in cell parameters are only used when they are defined by crystal symmetry. An approximate (isotropic) treatment of cell e.s.d.'s is used for estimating e.s.d.'s involving l.s. planes.
Refinement. Refinement of *F*^2^ against ALL reflections. The weighted *R*-factor *wR* and goodness of fit *S* are based on *F*^2^, conventional *R*-factors *R* are based on *F*, with *F* set to zero for negative *F*^2^. The threshold expression of *F*^2^ > σ(*F*^2^) is used only for calculating *R*-factors(gt) *etc*. and is not relevant to the choice of reflections for refinement. *R*-factors based on *F*^2^ are statistically about twice as large as those based on *F*, and *R*- factors based on ALL data will be even larger.

### Fractional atomic coordinates and isotropic or equivalent isotropic displacement parameters (Å^2^)

**Table:**

	*x*	*y*	*z*	*U*_iso_*/*U*_eq_	
C1	0.38673 (16)	0.3632 (2)	0.90220 (14)	0.0475 (5)	
C2	0.36836 (19)	0.5147 (3)	0.91678 (16)	0.0644 (6)	
C3	0.2891 (2)	0.5643 (3)	0.97699 (17)	0.0704 (7)	
C4	0.2262 (2)	0.4615 (3)	1.02507 (17)	0.0699 (7)	
C5	0.24340 (18)	0.3114 (3)	1.01251 (14)	0.0583 (6)	
C6	0.32381 (16)	0.2594 (2)	0.95135 (13)	0.0466 (5)	
C7	0.33731 (16)	0.0969 (2)	0.93682 (13)	0.0478 (5)	
C8	0.42994 (15)	0.0467 (2)	0.87708 (13)	0.0437 (5)	
C9	0.52038 (16)	0.1607 (2)	0.86272 (15)	0.0512 (5)	
C10	0.46780 (16)	0.3104 (2)	0.83054 (14)	0.0498 (5)	
C11	0.40543 (18)	0.3027 (3)	0.73028 (15)	0.0611 (6)	
C12	0.4811 (2)	0.2601 (4)	0.65044 (17)	0.0861 (9)	
C13	0.41996 (16)	−0.0882 (2)	0.83653 (13)	0.0482 (5)	
C14	0.49530 (16)	−0.1640 (2)	0.77224 (13)	0.0472 (5)	
C15	0.61293 (17)	−0.1592 (3)	0.78482 (15)	0.0598 (6)	
C16	0.6781 (2)	−0.2362 (3)	0.72341 (19)	0.0748 (8)	
C17	0.6287 (2)	−0.3177 (3)	0.64874 (17)	0.0734 (7)	
C18	0.5129 (2)	−0.3237 (3)	0.63530 (17)	0.0752 (7)	
C19	0.4468 (2)	−0.2491 (3)	0.69728 (16)	0.0652 (6)	
H10	0.5290	0.3845	0.8288	0.060*	
H11A	0.3445	0.2298	0.7315	0.073*	
H11B	0.3712	0.3998	0.7154	0.073*	
H12A	0.5471	0.3234	0.6539	0.129*	
H12B	0.4396	0.2730	0.5894	0.129*	
H12C	0.5041	0.1571	0.6579	0.129*	
H13	0.3561	−0.1426	0.8508	0.058*	
H15	0.6480	−0.1038	0.8350	0.072*	
H16	0.7569	−0.2327	0.7329	0.090*	
H17	0.6735	−0.3687	0.6074	0.088*	
H18	0.4787	−0.3781	0.5843	0.090*	
H19	0.3681	−0.2561	0.6885	0.078*	
H2	0.4106	0.5847	0.8851	0.077*	
H3	0.2779	0.6667	0.9853	0.085*	
H4	0.1724	0.4946	1.0657	0.084*	
H5	0.2011	0.2426	1.0450	0.070*	
H9A	0.5691	0.1247	0.8148	0.061*	
H9B	0.5667	0.1746	0.9222	0.061*	
O1	0.27360 (13)	0.00631 (18)	0.97155 (11)	0.0699 (5)	

### Atomic displacement parameters (Å^2^)

**Table:**

	*U*^11^	*U*^22^	*U*^33^	*U*^12^	*U*^13^	*U*^23^
C1	0.0455 (11)	0.0539 (13)	0.0434 (11)	0.0016 (9)	0.0058 (9)	0.0034 (9)
C2	0.0697 (14)	0.0580 (16)	0.0668 (15)	0.0008 (12)	0.0151 (12)	0.0043 (12)
C3	0.0805 (16)	0.0593 (15)	0.0728 (16)	0.0096 (13)	0.0141 (13)	−0.0093 (13)
C4	0.0745 (15)	0.0776 (19)	0.0604 (14)	0.0122 (13)	0.0237 (12)	−0.0098 (13)
C5	0.0609 (13)	0.0687 (16)	0.0477 (12)	0.0046 (11)	0.0202 (10)	0.0003 (11)
C6	0.0452 (11)	0.0569 (13)	0.0385 (11)	0.0031 (9)	0.0091 (9)	0.0007 (9)
C7	0.0460 (11)	0.0583 (14)	0.0406 (11)	−0.0012 (10)	0.0125 (9)	0.0046 (9)
C8	0.0390 (10)	0.0517 (13)	0.0415 (11)	0.0037 (9)	0.0108 (8)	0.0068 (9)
C9	0.0396 (10)	0.0578 (14)	0.0576 (13)	−0.0016 (9)	0.0132 (9)	0.0027 (10)
C10	0.0418 (10)	0.0553 (13)	0.0539 (12)	−0.0042 (9)	0.0145 (9)	0.0058 (10)
C11	0.0566 (12)	0.0771 (16)	0.0518 (13)	0.0029 (12)	0.0188 (10)	0.0121 (11)
C12	0.0870 (18)	0.117 (2)	0.0579 (15)	0.0054 (17)	0.0310 (14)	0.0036 (15)
C13	0.0418 (10)	0.0578 (14)	0.0464 (11)	−0.0009 (9)	0.0129 (9)	0.0064 (10)
C14	0.0487 (11)	0.0531 (13)	0.0410 (11)	0.0027 (9)	0.0111 (9)	0.0064 (9)
C15	0.0518 (12)	0.0729 (16)	0.0548 (13)	0.0125 (11)	0.0040 (10)	−0.0029 (11)
C16	0.0555 (13)	0.092 (2)	0.0790 (18)	0.0205 (13)	0.0213 (13)	−0.0014 (15)
C17	0.0875 (19)	0.0762 (18)	0.0607 (15)	0.0159 (14)	0.0341 (14)	0.0019 (13)
C18	0.0938 (19)	0.0823 (19)	0.0514 (14)	0.0004 (15)	0.0187 (13)	−0.0119 (13)
C19	0.0594 (13)	0.0794 (17)	0.0577 (14)	−0.0041 (12)	0.0104 (11)	−0.0069 (12)
O1	0.0714 (10)	0.0649 (10)	0.0790 (11)	−0.0055 (8)	0.0427 (9)	0.0053 (8)

### Geometric parameters (Å, °)

**Table:**

C1—C6	1.398 (3)	C11—H11B	0.9700
C1—C2	1.383 (3)	C11—H11A	0.9700
C2—H2	0.9300	C11—C12	1.528 (3)
C2—C3	1.376 (3)	C12—H12C	0.9600
C3—H3	0.9300	C12—H12B	0.9600
C3—C4	1.384 (3)	C12—H12A	0.9600
C4—H4	0.9300	C13—H13	0.9300
C5—H5	0.9300	C13—C14	1.474 (3)
C5—C4	1.365 (3)	C13—C8	1.330 (3)
C6—C5	1.402 (3)	C14—C15	1.387 (3)
C7—C8	1.492 (2)	C14—C19	1.383 (3)
C7—C6	1.471 (3)	C15—H15	0.9300
C7—O1	1.227 (2)	C15—C16	1.377 (3)
C8—C9	1.497 (3)	C16—H16	0.9300
C9—H9B	0.9700	C16—C17	1.367 (4)
C9—H9A	0.9700	C17—H17	0.9300
C10—H10	0.9800	C18—H18	0.9300
C10—C11	1.537 (3)	C18—C17	1.366 (4)
C10—C9	1.524 (3)	C19—H19	0.9300
C10—C1	1.511 (3)	C19—C18	1.379 (3)
			
C6—C1—C10	120.39 (18)	C18—C17—C16	119.5 (2)
C2—C1—C10	120.99 (18)	C19—C18—H18	120.0
C2—C1—C6	118.53 (18)	C17—C18—H18	120.0
C11—C10—H10	108.1	C17—C18—C19	120.1 (2)
C9—C10—H10	108.1	C14—C19—H19	119.4
C1—C10—H10	108.1	C18—C19—H19	119.4
C9—C10—C11	112.82 (18)	C18—C19—C14	121.2 (2)
C1—C10—C11	109.61 (16)	C1—C2—H2	119.2
C1—C10—C9	109.87 (16)	C3—C2—H2	119.2
H11A—C11—H11B	107.6	C3—C2—C1	121.6 (2)
C10—C11—H11B	108.7	C4—C3—H3	120.0
C12—C11—H11B	108.7	C2—C3—H3	120.0
C10—C11—H11A	108.7	C2—C3—C4	119.9 (2)
C12—C11—H11A	108.7	C3—C4—H4	120.2
C12—C11—C10	114.33 (19)	C5—C4—H4	120.2
H12B—C12—H12C	109.5	C5—C4—C3	119.6 (2)
H12A—C12—H12C	109.5	C6—C5—H5	119.5
C11—C12—H12C	109.5	C4—C5—H5	119.5
H12A—C12—H12B	109.5	C4—C5—C6	121.0 (2)
C11—C12—H12B	109.5	C5—C6—C7	119.61 (18)
C11—C12—H12A	109.5	C1—C6—C7	121.03 (17)
C14—C13—H13	115.3	C1—C6—C5	119.3 (2)
C8—C13—H13	115.3	C6—C7—C8	117.57 (16)
C8—C13—C14	129.46 (18)	O1—C7—C8	121.35 (19)
C15—C14—C13	123.46 (19)	O1—C7—C6	121.07 (17)
C19—C14—C13	118.62 (18)	C7—C8—C9	115.53 (17)
C19—C14—C15	117.88 (19)	C13—C8—C9	126.63 (17)
C14—C15—H15	119.8	C13—C8—C7	117.63 (17)
C16—C15—H15	119.8	H9A—C9—H9B	108.1
C16—C15—C14	120.4 (2)	C10—C9—H9B	109.5
C15—C16—H16	119.5	C8—C9—H9B	109.5
C17—C16—H16	119.5	C10—C9—H9A	109.5
C17—C16—C15	120.9 (2)	C8—C9—H9A	109.5
C16—C17—H17	120.3	C8—C9—C10	110.72 (16)
C18—C17—H17	120.3		
